# A Durum Wheat Variety-Based Product Is Effective in Reducing Symptoms in Patients with Non-Celiac Gluten Sensitivity: A Double-Blind Randomized Cross-Over Trial

**DOI:** 10.3390/nu11040712

**Published:** 2019-03-27

**Authors:** Gianluca Ianiro, Gianenrico Rizzatti, Marco Napoli, Maria Valeria Matteo, Emanuele Rinninella, Vincenzina Mora, Caterina Fanali, Alessia Leonetti, Stefano Benedettelli, Maria Cristina Mele, Giovanni Cammarota, Antonio Gasbarrini

**Affiliations:** 1 Digestive Disease Center, Fondazione Policlinico Universitario A. Gemelli IRCCS - Università Cattolica del Sacro Cuore, 00143 Rome, Italy; gianluca.ianiro@hotmail.it (G.I.); gianenrico.rizzatti@gmail.com (G.R.); marco.napoli.md@gmail.com (M.N.); mariavaleria31191@gmail.com (M.V.M.); emanuele.rinninella@unicatt.it (E.R.); vincenzina.mora@gmail.com (V.M.); caterinafanali@gmail.com (C.F.); alessia.leonetti1@gmail.com (A.L.); MariaCristina.Mele@unicatt.it (M.C.M.); Giovanni.Cammarota@unicatt.it (G.C.); Antonio.Gasbarrini@unicatt.it (A.G.); 2 Dipartimento di Scienze e Tecnologie Agrarie, Alimentari, Ambientali e Forestali (DAGRI), Università degli Studi di Firenze, 50144 Firenze, Italy

**Keywords:** non-celiac gluten sensitivity, gluten, gluten-free diet, wheat

## Abstract

Patients with non-celiac gluten sensitivity (NCGS) do not have celiac disease, but their symptoms improve after a gluten-free diet (GFD). However, to date, it is uncertain if gluten or other components of wheat are responsible for these symptoms. The aim of this study was to compare the effects of an organic durum wheat variety with those of standard commercial wheat in patients with known NCGS. We performed a double-blind randomized cross-over trial of 42 patients (mean age 45 years, 8 men) with NCGS diagnosed according to the Salerno criteria and adherence to GFD for at least 12 weeks from screening. Enrolled subjects were randomly assigned to one the following groups of treatment: (A) a two-week diet with Senatore Cappelli wheat variety pasta; (B) a two-week diet with standard commercial pasta. Then, after a two-week washout period on gluten-free diet, each patient crossed over to the other treatment group. Symptoms were assessed through a modified version of the Gastrointestinal Symptom Rating Scale (GSRS), tailored on NCGS. Between April 2018 and July 2018, 42 patients with NCGS were enrolled in the study (70.6% females), and 34 patients completed the study. Patients reported lower overall symptoms scores after eating Senatore Cappelli pasta than standard pasta (*p* = 0.03) and also significantly lower scores in several specific gastrointestinal and extra-intestinal symptoms after eating Senatore Cappelli pasta than standard pasta, specifically, bloating (*p* = 0.04), abdominal distention (*p* = 0.004), eructation (*p* = 0.01), flatus (*p* = 0.02), feeling of incomplete evacuation (*p* = 0.001), dermatitis (*p* = 0.01), and limb numbness (*p* = 0.03). In our study, patients with NCGS experienced lower gastrointestinal and extra-intestinal symptom scores after eating the Senatore Cappelli wheat variety than a standard commercial wheat. Should our preliminary results be confirmed by further studies, new dietary alternatives may be available to patients with NCGS, with consequent health, economic, and social benefits.

## 1. Introduction

Several gastrointestinal disorders are elicited by the consumption of specific dietary components, the most common being dietary allergies, lactose intolerance, and gluten-related disorders [1], which include celiac disease (CD), wheat allergy, and non-celiac gluten sensitivity (NCGS).

NCGS is a complex syndrome characterized by the occurrence of intestinal and extra-intestinal symptoms related to the consumption of gluten-containing foods in subjects in which CD and wheat allergy have been excluded by an appropriate work-up [2]. While the mainstay of CD management consists in a strict adherence to a gluten-free diet (GFD) [3], the role of gluten in the pathogenesis of NSCG has not been yet clarified. While avoidance of gluten is frequently advised in NCGS, evidence supporting this practice is low and often conflicting [4,5,6]. In a double-blind cross-over randomized trial of patients with self-reported NCGS, gluten did not exert any effect after dietary reduction of fermentable, poorly absorbed, short-chain carbohydrates [5]. In another double-blind randomized clinical trial, only 14% of patients whose symptoms improved after a three-week-long GFD showed a symptomatic relapse during a further gluten challenge [6]. 

On the basis of this evidence, other components of wheat, including agglutinins, fructans, or amylase trypsin inhibitors, are thought to play a role in the pathogenesis of this condition [7,8,9,10]. Accordingly, the term “non-celiac wheat sensitivity” (NCWS) is increasingly being preferred to that of NCGS [11,12,13].

Moreover, a strict GFD might be associated with nutritional and metabolic abnormalities, such as increased caloric intake, low consumption of fibers, possibly, micro- and macronutrients deficiencies [1], and alterations in the gut microbiota, including a reduction in the abundance of beneficial bacteria [14]. Finally, the consumption of gluten-free products could be particularly expensive for these patients.

For these reasons, the identification of more tolerable wheat varieties [15] for patients with NCGS/NCWS could be extremely interesting, as it may represent a more suitable dietetic alternative to GFD with less detrimental effects on nutritional status, being also cheaper.

Among different wheat varieties, the Senatore Cappelli one is an Italian traditional strain of durum wheat which displays more favorable nutritional characteristics, including a higher content of fibers and micronutrients and reduced pesticide contamination, such as glyphosate [15,16,17,18].

The aim of the present study was to compare the effects of Senatore Cappelli wheat variety with those of a commercially available wheat product in subjects with NCGS/NCWS.

## 2. Methods

### 2.1. Patients

All patients attending the outpatient gastroenterology clinic of the Fondazione Policlinico Universitario “A. Gemelli” IRCCS from March 2018 to July 2018 were evaluated for recruitment. The inclusion criteria were: older than 18 years of age; diagnosis of NCGS according to the Salerno criteria (19); absence of symptoms while on GFD; adherence to GFD for at least 12 weeks from screening. Exclusion criteria were: paediatric age; diagnosis of celiac disease or wheat allergy; pregnancy or breastfeeding; relevant comorbidities (e.g., inflammatory bowel disease, liver cirrhosis, etc.); impossibility to adhere to the procedures of the study protocol. All enrolled subjects provided their written informed consent. The study protocol was approved by the ethics committee of the Fondazione Policlinico Universitario “A. Gemelli” IRCCS.

### 2.2. Study Design

This study was designed as a double-blind randomized, controlled, cross-over trial. At baseline, all subjects underwent a medical and nutritional visit, where they recorded their symptoms and dietary habits and were given a dietary plan to be followed for the whole study period. Afterwards, they were randomly assigned to one the following groups of treatment: (A) a two-week diet with pasta obtained from Senatore Capelli durum wheat variety; (B) a two-week diet with standard commercial pasta. 

Subsequently to the completion of this first period, all subjects were put back on GFD for a two-week washout period. Then, each patient crossed over to the other treatment group for two more weeks. All subjects were asked to fill a daily symptom questionnaire and a dietary adherence questionnaire for the whole study period. Patients were asked to describe extensively any additional food eaten out of the dietary prescription and to bring back the pasta boxes that were not consumed.

Gastrointestinal symptoms were assessed by the participant completing daily diary cards using a modified version of the Gastrointestinal Symptom Rating Scale (GSRS), tailored on NCGS (19). This scale includes the evaluation of intestinal as well as extra-intestinal symptoms, and up to 10 points can be assigned for each item on a visual analog scale (VAS).

If patients were unfit to continue a specific diet because of intolerable symptoms, they stopped the treatment and the collection of symptom scores, were put on a gluten-free diet until the completion of the period, and received the remaining treatment, if any, after the washout period. 

Patients underwent visits by the gastroenterology team (G.I., G.R., M.N., M.V.M., A.G.) and by the dietician team (E.R., M.C.M.) at baseline and at the end of each two-week period. Moreover, dieticians followed patients weekly by phone visits to check their adherence to diet.

### 2.3. Study Food Characteristics

Durum wheat (*Triticum turgidum* L. subsp. *durum*) Senatore Cappelli variety was used to obtain the experimental pasta. A standard, commercially available semolina pasta, obtained from different variety of grains originating from Italy and other European and non-European countries and milled in Italy, was used as comparator. Both pasta types were provided by the Società Italiana Sementi (S.I.S) Bologna (Italy). The daily amount of pasta that patients were requested to consume was 100 g. Packages of both types of pasta were identical and did not permit to recognize the type of pasta. The packages were shipped to our center by S.I.S in different boxes. The unblinded team prepared the single packages of pasta by labelling them. The label reported the name of the principal investigator, the affiliated department, and general indications on how to prepare and store the pasta. On the label, the letter A or B allowed the unblinded team to distinguish the two types of pasta. Neither investigators nor patients were aware of the association between letters and types of pasta.

### 2.4. Outcomes

The primary end point was the difference in overall symptom score, measured on a VAS, between the two treatments.

Secondary end points were differences in each individual symptom score, measured on a VAS at the end of each treatment period, between the two treatments.

### 2.5. Randomization

The randomization of the subjects was performed by a member of the unblinded team. The generator software, provided by www.sealedenvelope.com, generated random permuted blocks, setting a block size of 10 and an equal allocation ratio. The randomization list was kept sealed for the investigators until the end of the study; only the unblinded team was authorized to open the envelope to assign the correct boxes of pasta to the patients.

### 2.6. Statistical Analysis

This randomized controlled trial was conceived as a proof of concept, as there are no data suggesting how much the consumption of Senatore Cappelli pasta changed symptom scores compared with standard commercial pasta in patients with NCGS. Following previous similar studies [4,5], we decided to enroll 21 patients per arm.

Per-protocol analysis was performed. Only patients who entered both study treatments were considered for the analysis. Wilcoxon signed-rank test was used to compare nonparametric data. The Bonferroni correction for multiple comparisons was applied. Two-tailed p values <0.05 were considered statistically significant. Statistical analyses were carried out with an online calculator (http://www.graphpad.com/quickcalcs/) and with Microsoft Excel for Mac (Microsoft Excel. Redmond, WA, USA: Microsoft, 2011).

## 3. Results

### 3.1. Study Population

Between April 2018 and July 2018, 116 patients referred to the Digestive Disease Center of the Fondazione Policlinico “A. Gemelli” IRCCS were assessed for eligibility. Seventy-four subjects were excluded for the following reasons: 52 patients did not fulfil the inclusion criteria, 16 patients were inadequately adherent to GFD at baseline, and 16 patients were unwilling to participate. The remaining 42 subjects were included in the study. Their demographic and clinical characteristics at baseline are detailed in Table 1.

### 3.2. Adherence to the Study Treatments

Thirty-four patients (81%) received both study treatments, while eight patients (19%) ceased the study prematurely because of intolerable symptoms and refused to assume the other treatment. Regarding these eight patients, five had taken the standard commercial pasta, and three the Senatore Cappelli pasta. All of them dropped out of the study on average after three days of consumption of the first dietary treatment. 

Among the 34 patients who completed both study treatments, 30 completed the two-week intake of the Senatore Cappelli pasta, and 25 completed the two-week intake of standard commercial pasta. Three patients withdrew the consumption of Senatore Cappelli pasta after, respectively, 4, 7, and 8 days of intake. Nine patients stopped eating the standard commercial pasta after, respectively, 1, 2, 4, 5, 7, 10, 10, 11, and 11 days of intake. All patients adhered to the prescribed dietary schedule during the study. The overall duration of our study was five months (April–September 2018).

### 3.3. Effect on Gastrointestinal and Extra-Intestinal Symptoms

**Figure 1:**
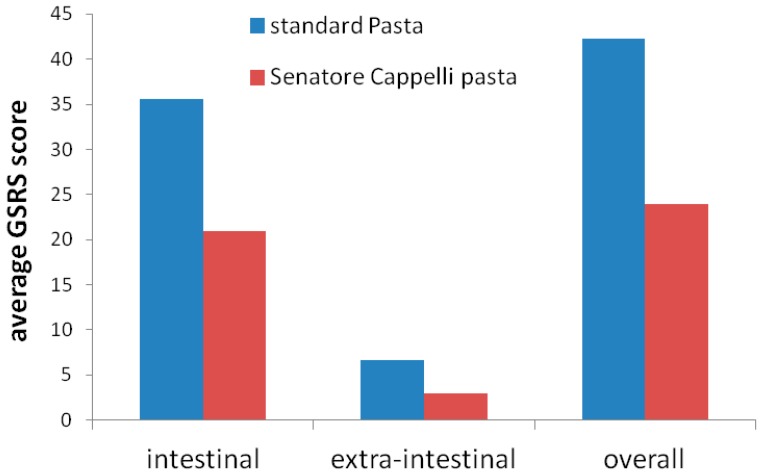
Average Gastrointestinal Symptom Rating Scale (GPRS) scores for intestinal, extra-intestinal, and overall symptoms in standard and Senatore Cappelli pasta groups.

Patients reported significantly lower overall and intestinal symptoms scores after eating Senatore Cappelli pasta than standard pasta (*p* = 0.03 and *p* = 0.02, respectively). The average GSRS scores for intestinal, extra-intestinal, and overall symptoms are reported in Figure 1.

**Figure 2:**
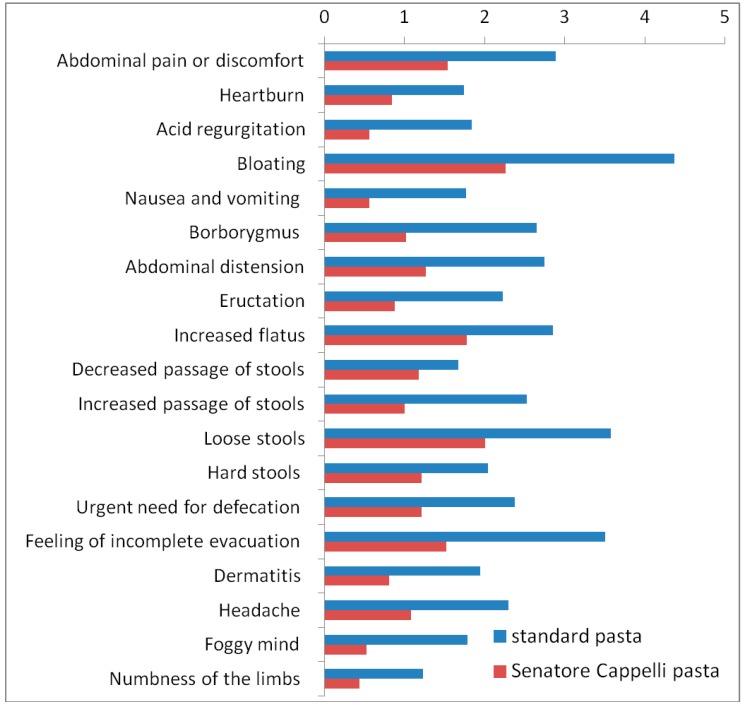
Scores for each item of the GSRS questionnaire.

Figure 2 shows symptom scores for each item of the GSRS questionnaire (Data can be found in Table S1). Patients experienced significantly lower scores in several specific gastrointestinal and extra-intestinal symptoms after eating Senatore Cappelli pasta than standard pasta. Specifically, lower scores in bloating (average 3.99 vs. 2.26; *p* = 0.04), abdominal distention (average 2.75 vs. 1.27; *p* = 0.004), eructation (average 2.23 vs. 0.88; *p* = 0.01), flatus (average 2.85 vs. 1.78; *p* = 0.02), and feeling of incomplete evacuation (average 3.5 vs. 1.52; *p* = 0.001) were reported after consumption of Senatore Cappelli pasta compared with standard pasta. 

Likewise, patients experienced significantly lower scores in extra-intestinal symptoms, including dermatitis (average 1.94 vs. 0.81; *p* = 0.01) and limb numbness (average 1.23 vs. 0.44; *p* = 0.03) after eating Senatore Cappelli pasta than standard pasta. Though not statistically significant, a trend toward lower scores in number of evacuation (*p* = 0.05), nausea, vomit, bowel sound (*p* = 0.06), loose stools evacuation (*p* = 0.07), acid regurgitation, and headache (*p* = 0.08) was observed after eating Senatore Cappelli pasta. After the Bonferroni correction, a significant difference in feeling of incomplete evacuation (*p* = 0.019) and a trend toward reduced abdominal distension (*p* = 0.076) were found.

No adverse events were reported in both treatment arms.

## 4. Discussion

Although NCGS is becoming increasingly popular, its pathogenesis is still poorly understood. An established body of knowledge suggests that gluten is the main dietary trigger of NCGS [19,20,21].

However, recent evidence has downsized the role of gluten in NCGS, as only a minority of patients have been found to experience recurrence of symptoms after blinded administration of gluten [22]. For this reason, other wheat components are suspected of playing a role in the development of symptoms associated with NCGS, including alpha amylase and trypsin inhibitors [23], fermentable, poorly absorbed, short-chain carbohydrates (fermentable, oligo-, di-, monosaccharides, and polyols (FODMAPs) [5] and, more recently, fructans [8]. 

In this double-blind randomized cross-over clinical trial, we found that patients with NCGS reported lower overall and specific extra-intestinal and gastrointestinal symptoms scores after eating pasta made with Senatore Cappelli wheat variety than standard pasta (*p* = 0.03). 

The Senatore Cappelli wheat variety has specific characteristics that could explain the lower symptom scores reported by patients. The Senatore Capelli durum wheat variety, derived from a pure line selection of a Tunisian Jeann Rhetifah ecotype, is an ancient organic wheat single variety, while the standard commercial comparator pasta was a blend of different grains cultivated at intensive levels. The Senatore Cappelli variety displays significantly higher amounts of secondary metabolites, including free and bound polyphenol isomers, than other wheats [24]. Drastic technological processes applied to wheat, including high refining, have been hypothesized to make wheat-based products less digestible through the loss of antioxidant and/or anti-inflammatory compounds and then to drive to NCGS-related symptoms [25,26]. Additionally, the properties of Senatore Cappelli variety were shown to be less influenced by environmental conditions than other varieties, and this may affect gliadin content and consequent immunogenic potential [27]. As both formulations of pasta included gluten and the Senatore Cappelli variety displays a higher protein content in the caryopsis (14–15% on a dry weight basis) than more recent varieties, our study confirms previous data that support the role of other non-gluten components in the development of symptoms associated with NCGS. As already suggested by other authors, the term NCWS seems to be, therefore, more appropriate than NCGS, as more than a single wheat component is reasonably implicated in the development of symptoms associated with the disease [28].

Our results also suggest that patients with NCGS may consume a specific durum wheat variety, even if with gluten, with several potential benefits, including the avoidance of nutritional and/or metabolic deficiencies [1], of gut microbiota imbalance [14], and of high expenses for gluten-free products. However, our study was not designed to assess microbiota and/or nutritional changes after treatment, neither was a cost analysis performed. Therefore, further studies specifically designed to assess these outcomes are needed.

Our study displays several strengths. First, we applied a cross-over design with a washout period that reduces carry-over and order effects and has already been used in similar previous studies [5,22,29,30]. Moreover, as the recent Salerno consensus conference suggests [31], we assessed outcomes through a modified version of the GSRS that, beside gastrointestinal symptoms, also includes the evaluation of extra-intestinal symptoms, which are usually also present in NCGS [31,32].

Finally, a careful nutritional assessment, including evaluation of dietary adherence, was performed by expert nutritionists at every study phase, and all patients were given a specific dietary schedule to reduce diet-related confounding factors.

However, our study has also some limitations. The diagnosis of NCGS was performed by applying the Salerno criteria, and CD and wheat allergy were excluded. CD was excluded on the basis of negative transglutaminase and endomysium antibody results in the presence of normal total serum IgA, while data on HLA status and duodenal histology were not available for all patients. Moreover, regular evaluation of CD serology was not performed throughout the study. Another limitation is the absence of symptom scores at baseline and after the washout period, as the presence of symptoms during these two phases of the study could have influenced the results. However, we included only patients with a diagnosis of NCGS who were asymptomatic at baseline, when under GFD. Finally, we acknowledge that this is a pilot study, without any sample size calculation, because data on the effects of Senatore Cappelli durum wheat variety on symptoms related to NCGS were not available at the beginning of the study. However, we decided to enroll 21 patients per arm on the basis of previous similar studies [4,5]. These results will allow to design larger studies with an appropriate sample size. 

To conclude, in our study, patients with NCGS experienced lower gastrointestinal and extra-intestinal symptom scores after eating the Senatore Cappelli wheat variety than a standard commercial wheat. Should our preliminary results be confirmed by further studies, new dietary alternatives may be available to patients with NCGS, with consequent health, economic, and social benefits.

## Figures and Tables

**Table 1:** Patients’ demographic and clinical characteristics at baseline.

Characteristics	
Sex, F (%)	24 (70.6%)
Age (average ± SD)	42.5 ± 13.4 years
**Elevated serum celiac antibodies (percentage of patients)**	
Tissue transglutaminase (IgA)	0
Tissue transglutaminase (IgG)	0
Endomysium (IgA)	0

## Supplementary Materials

The following are available online at https://www.mdpi.com/2072-6643/11/4/712/s1, Table S1: scores for each item of the GSRS questionnaire.
