# Supplementary material for: A Durum Wheat Variety-Based Product Is Effective in Reducing Symptoms in Patients with Non-Celiac Gluten Sensitivity: A Double-Blind Randomized Cross-Over Trial
Source: Nutrients. 2019 Mar 27;11(4):712. doi: 10.3390/nu11040712 (PMC6521061; doi:10.3390/nu11040712)
Supplement: Supplementary file 1 [file nutrients-11-00712-s001.pdf]

**Supplementary Table 1.** scores for each item of the GSRS questionnaire

| | Commercial | Durum Wheat | P value (Wilcoxon) |
|---|---|---|---|
| **INTESTINAL SYMPTOMS** | | | |
| Abdominal pain or discomfort | 2.89 | 1.54 | 0.17 |
| Heartburn | 1.74 | 0.84 | 0.31 |
| Acid regurgitation | 1.84 | 0.56 | 0.08 |
| Bloating | 4.37 | 2.26 | **0.04** |
| Nausea and vomiting | 1.77 | 0.56 | 0.06 |
| Borborygmus | 2.65 | 1.02 | 0.06 |
| Abdominal distension | 2.75 | 1.27 | **0.004** |
| Eructation | 2.23 | 0.88 | **0.01** |
| Increased flatus | 2.85 | 1.78 | **0.02** |
| Decreased passage of stools | 1.67 | 1.18 | 0.1 |
| Increased passage of stools | 2.53 | 1.00 | 0.05 |
| Loose stools | 3.57 | 2.01 | 0.07 |
| Hard stools | 2.04 | 1.21 | 0.1 |
| Urgent need for defecation | 2.38 | 1.21 | 0.2 |
| Feeling of incomplete evacuation | 3.5 | 1.52 | **0.001** |
| **EXTRA-INTESTINAL SYMPTOMS** | | | |
| Dermatitis | 1.94 | 0.81 | **0.01** |
| Headache | 2.30 | 1.08 | 0.08 |
| Foggy mind | 1.79 | 0.53 | 0.12 |
| Numbness of the limbs | 1.23 | 0.44 | **0.03** |
